# A Prognostic Model Based on the Immune-Related lncRNAs in Colorectal Cancer

**DOI:** 10.3389/fgene.2021.658736

**Published:** 2021-04-20

**Authors:** Fengxia Qin, Houxi Xu, Guoli Wei, Yi Ji, Jialin Yu, Canhong Hu, Chunyi Yuan, Yuzhu Ma, Jun Qian, Lingchang Li, Jiege Huo

**Affiliations:** ^1^Affiliated Hospital of Integrated Traditional Chinese and Western Medicine, Nanjing University of Chinese Medicine, Nanjing, China; ^2^Key Laboratory of Acupuncture and Medicine Research of Ministry of Education, Nanjing University of Chinese Medicine, Nanjing, China; ^3^Department of Oncology, Ganyu District Hospital of Traditional Chinese Medicine, Lianyungang, China; ^4^School of Chinese Medicine, Nanjing University of Chinese Medicine, Nanjing, China

**Keywords:** colorectal cancer, immune-related lncRNA, prognostic index, bioinformatics, TCGA

## Abstract

**Background:**

Colorectal cancer (CRC) is one of the most common malignant tumors with a poor prognosis. At present, the pathogenesis is not completely clear. Therefore, finding reliable prognostic indicators for CRC is of important clinical significance. In this study, bioinformatics methods were used to screen the prognostic immune-related lncRNAs of CRC, and a prognostic risk scoring model based on immune-related lncRNAs signatures were constructed to provide a basis for prognostic evaluation and immunotherapy of CRC patients.

**Methods:**

The clinical information and RNA-seq data of CRC patients were obtained from The Cancer Genome Atlas (TCGA) database. The information of immune-related lncRNA was downloaded from the immunology database and analysis portal. The differentially expressed immune-related lncRNAs (IRLs) were screened by the edgeR package of R software. The prognostic value of IRLs was studied. Based on Cox regression analysis, a prognostic index (IRLPI) based on IRLs was established, and the relationship between the risk score and the clinicopathological characteristics of CRC was analyzed to determine the effectiveness of the risk score model as an independent prognostic factor.

**Results:**

A total of 240 differentially expressed IRLs were identified between normal colorectal cancer tissues and normal colorectal cancer tissues, in which 8 were significantly associated with the survival of CRC patients (*P* < 0.05), including *LINC00461, LINC01055, ELFN1-AS1, LMO7-AS1, CYP4A22-AS1, AC079612.1, LINC01351*, and *MIR31HG*. And most of the lncRNAs related to survival were risk factors for the prognosis of CRC. The index established based on the 7 survival-related IRLs found to be highly accurate in monitoring CRC prognosis. Besides, IRLPI was significantly correlated with a variety of pathological factors and immune cell infiltration.

**Conclusion:**

Eight immune-related lncRNAs closely related to the prognosis of CRC patients were identified from the TCGA database. At the same time, an independent IRLPI was constructed, which may be helpful for clinicians to assess the prognosis of patients with CRC and to formulate individualized treatment plans.

## Introduction

Colorectal cancer (CRC) is the third most common malignant tumor in the world (Arnold et al., 2017). More than half of the patients suffered recurrence and distant metastasis at the time of diagnosis and during the follow-up treatments (Huiskens et al., 2015). Although traditional surgery, chemotherapy, radiotherapy, and targeted therapy could extend the median survival of patients appropriately, CRC is still the fourth leading cause of cancer-related deaths with a poor prognosis (Miller et al., 2019). In recent years, immunotherapy had shown good efficacy in clinical trials such as leukemia (Xu and Niu, 2020) and non-small cell lung cancer (Wu et al., 2021). Related trials have also been carried out in CRC to change the traditional treatment model (Forster and Radpour, 2020). Unfortunately, immunotherapy works only for some patients, so it remains a challenge to identify patients who are effective for specific drugs as soon as possible. Moreover, tumor-induced immune escape is common, and many problems need to be solved in immunotherapy, especially in the field of identifying immunotherapy biomarkers and new effective therapeutic targets. In the past decade, advances in genome sequencing and analysis have made the understanding of the CRC genome and immune microenvironment increased (Wen et al., 2020), individualized therapy can be carried out based on the specific genomic characteristics of CRC, leading to the development of precision medicine. Therefore, it is urgent to explore effective prognostic biomarkers and predictive models for facilitating and evaluating the diagnosis, the molecular mechanism, the immune microenvironment and treatment of CRC.

Long non-coding RNAs (lncRNAs), which are more than 200 nucleotides in length, are RNA transcripts that do not code for proteins. LncRNAs played extensive regulatory roles in different stages of tumor immune response, such as antigen exposure, antigen recognition, immune activation, immune cell infiltration, tumor clearance, and so on (Flores-Concha and Oñate, 2020). A study showed that *Lnc-LALC* can regulate gene expression levels and promote liver metastasis by regulating gene methylation status in colorectal cancer (Zhang et al., 2021). Another study suggested that lncRNA *SATB2-AS1* was specifically expressed in colorectal tissues and survival analysis indicated that decreased *SATB2-AS1* expression was associated with poor survival (Xu M. et al., 2019). The expression of lncRNA *MIR17HG* was gradually upregulated in colorectal cancer tissue and can promote tumorigenesis and metastasis in CRC cells both *in vitro* and *in vivo*. *MIR17HG* also upregulated PD-L1, indicating its potential role in immunotherapy (Xu J. et al., 2019). These studies indicated that the expression levels of lncRNA affect the development and prognosis of colon cancer.

Considering the above factors, we tried to use immune-related lncRNA to construct a risk assessment model to assess the survival and prognosis of patients with colorectal cancer and optimize clinical treatment decisions. In this study, we identified the differentially expressed IRLs in CRC patients by correlating gene expression profiles to IRLs from different online databases firstly. Then the prognostic value of the survival-related IRLs was also determined. Finally, the prognostic indicators were established based on Cox regression analysis, and the model was evaluated and applied. The obtained prognostic biomarkers could be used to predict the clinical outcome of treatments including cancer immunotherapy.

## Materials and Methods

### Data Download

The RNA-sequencing data of CRC samples were downloaded from The Cancer Genome Atlas (TCGA) data portal (Tomczak et al., 2015), and the clinical information was obtained by Perl script. In total 644 tumor tissues and 51 normal tissues were included. Furthermore, a comprehensive list of immune-related lncRNAs (2617 in total) of colorectal cancer was downloaded from ImmLnc (Li Y. et al., 2020). ImmLnc is a web-based resource for investigating the immune-related function of lncRNAs across cancer types. In the resource, the users can query the lncRNA-pathways, lncRNA-immune cell type’s correlation, and cancer-related lncRNAs across 33 cancer types.

### Identification of Differentially Expressed lncRNAs

The DElncRNAs were identified by comparing the paired CRC and adjacent normal tissues using the edgeR package of R software (Varet et al., 2016). The screening criteria for DElncRNAs were: | log2(fold change)| >2 and false discovery rate (FDR) <0.05. Then, the differentially expressed IRLs were extracted from the list of identified DElncRNAs. The heatmap and volcano plot of DElncRNA were generated by the heatmap package and ggplot2 package of R software, respectively (Gu et al., 2016; Maag, 2018).

### Screening of Survival-Related IRLs and Evaluation of the Prognostic Value

Survival analysis of all the differentially expressed IRLs was obtained by using the survival package of R software (Groeneveld et al., 2019), and the survival-related IRLs were dependent on univariate Cox analysis (*P* < 0.05). To define the prognostic value of survival-related IRLs, a forest plot of these lncRNAs was created by using the hazard ratio (HR) as an indicator, relied on univariate Cox analysis.

### Development of the IRLs-Based Prognostic Index

Survival-related IRLs were referred to multivariate analysis to develop the IRLPI, and completed IRLs were regarded as independent prognostic factors. In particular, the IRLPI was generated by multiplying the expression values with the Cox regression coefficient.

### Assessment of IRLPI and Mutation Analysis

Patients were divided into two groups according to the median value of risk scores. If IRLPI was higher than the median, it was assigned to the high-risk group; otherwise, it was assigned to the low-risk group. To prove the Overall Survival (OS) of patients in different risk groups, the homologous Kaplan-Meier (Kmurm) survival curve was drawn. To evaluate the specificity and sensitivity of the model, the ROC curve was constructed. Besides, the survival package of R software was used to conduct a univariate and multivariate analysis of survival rates for IRLPI and clinicopathological factors (Li T. et al., 2020). At the same time, the correlation between the expression of IRLs and clinicopathological factors was explored. Additionally, the genetic variation analysis of IRLs was analyzed by using the cBioPortal website.

### Evaluation of Immune Cell Infiltration in Cancers

The Tumor Immune Estimation Resource (TIMER) is a resource for systematical evaluations of the clinical impact of different immune cells in multiple cancer types (Li Y. et al., 2020). Statistical methods were used to calculate the abundances of six immune cell types in the tumor microenvironment, including B cells, CD4 T cells, CD8 T cells, neutrophils, macrophages, and dendritic cells. The obtained results had been validated using pathological estimations. Then, the levels of immune cell infiltration in CRC samples were downloaded, and the association between IRLPI and immune cell infiltration was calculated by Pearson correlation analysis.

### Statistical Analysis

To evaluate the effectiveness of IRLPI, the survivalROC package of R software was adopted to calculate the area under the curve (AUC) of the receiver operating characteristic (ROC) curve (Saha-Chaudhuri and Heagerty, 2013). An independent *t*-test was used to exam the differences among different clinical parameters. If *P* < 0.05, these differences were considered to be statistically significant.

### Verification of IRLs in IRLPI

The basis of the IRLPI model is that there are significant differences in the relative expression of IRLs. To verify the reliability of IRLs in IRLPI, we collected ten clinical samples of colorectal cancer patients, including colorectal cancer tissues and colorectal normal tissues, and then used the quantitative real-time PCR (qRT-PCR) method for molecular verification. We extracted total RNA from the tissues using TRIzol reagent. Total RNA was reverse transcribed into cDNA using a PrimeScript RT reagent kit with gDNA Eraser (Takara, Dalian, China). The primers were designed using Primer BLAST. The expression levels of lncRNAs were measured using a real-time PCR system (Applied Biosystems, Foster City, CA, United States). *GAPDH* was used as the internal reference gene. Finally, we analyzed the data by the Comparative Threshold (CT) (2^–ΔΔCT^) method. The research was approved by the Ethics Committee of the Jiangsu Provincial Hospital of Integrated Traditional Chinese and Western Medicine. All patients provided written informed consent for research on their specimens. For detailed information regarding qRT-PCR primers of lncRNAs, please refer to Supplementary Table 1.

## Results

### Identification of Differentially Expressed IRLs

**FIGURE 1:**
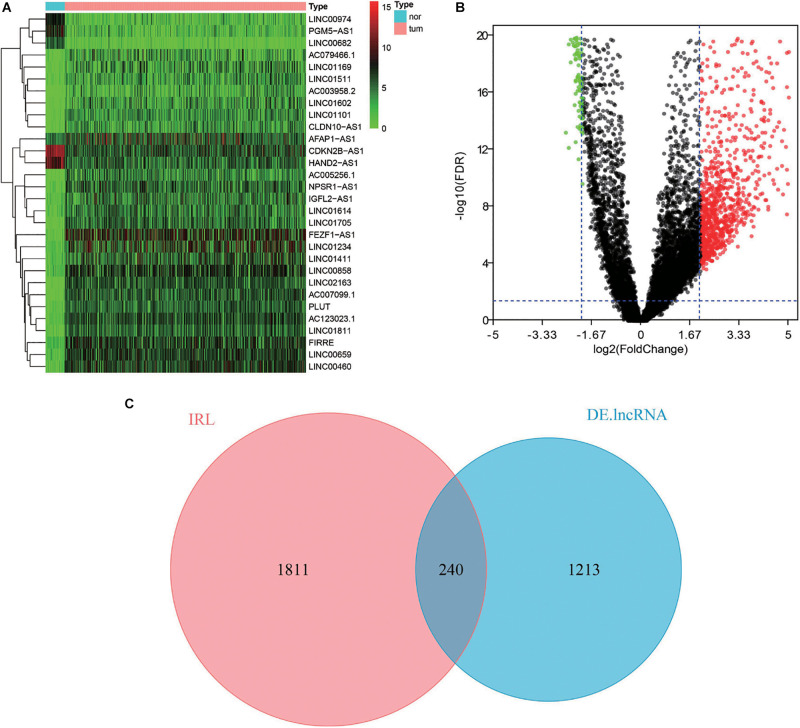
Differential expression analysis of IRLs. **(A)** Heatmap of top 30 DElncRNAs between normal tissues and CRC tissues. The higher and lower expressed lncRNAs were shown in red and green, respectively, and lncRNAs with the same expression level in black. **(B)** Volcano plot of DElncRNAs between normal tissues and CRC tissues. The red dots represent up-regulated DEIncRNAs, while the green dots represent down-regulated DEIncRNAs. **(C)** Venn diagram of differentially expressed IRLs. The two circles in the figure represent IRLs and DElncRNAs, respectively, and the purple area in the middle represents the differentially expressed IRLs.

To identify the DElncRNAs, the edgeR package of R software was used to compare the lncRNAs data of 644 CRC samples and 51 adjacent normal samples. According to the cutoff criteria of | log2(fold change)| >2 and FDR <0.05, a total of 1,453 DElncRNAs were detected, containing 1,000 up-regulated lncRNAs and 453 down-regulated lncRNAs (Figures 1A,B). With the IRLs list, 240 differentially expressed IRLs were extracted from all DElncRNAs (Figure 1C), including 100 up-regulated IRLs and 140 down-regulated IRLs.

### Acquisition of Survival-Related IRLs

As a governing prognostic factor for clinical tumor monitoring applications, molecular biomarkers relevant to IRLs can be used to evaluate the efficacy of tumor immunotherapy. Univariate Cox analysis showed that eight survival-related IRLs were significantly different (*P* < 0.05), including *LINC00461, LINC01055, ELFN1-AS1, LMO7-AS1, CYP4A22-AS1, AC079612.1, LINC01351*, and *MIR31HG*.

### Prognostic Values of Survival-Related IRLs

**FIGURE 2:**
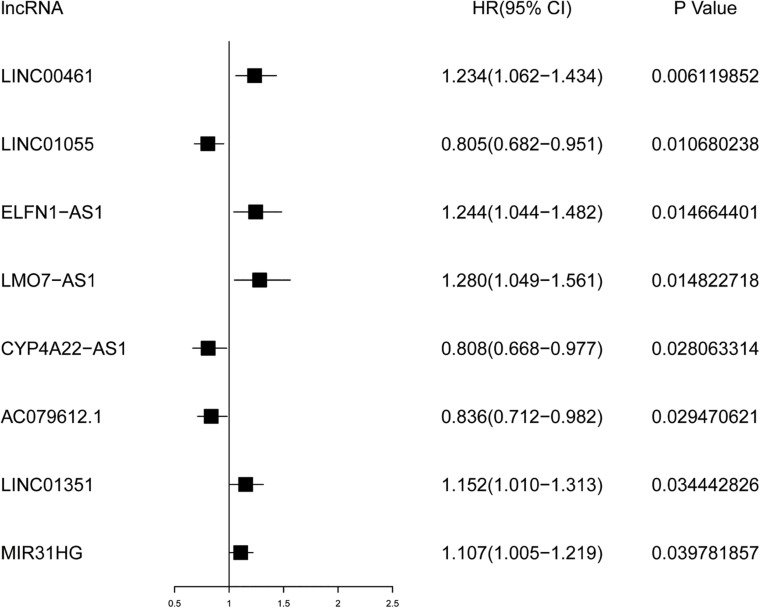
Forest plot for survival analysis of IRLs based on univariate Cox regression analysis. *P* < 0.05 is supposed to be statistically significant. HR >1 represents the adverse prognostic factor, and HR <1 represents the favorable prognostic factor.

The prognostic values of these lncRNAs were calculated to determine the characteristics of survival-related IRLs. The results demonstrated in Figure 2 showed that 5 lncRNAs were marked by HR >1 and 3 lncRNAs were marked by HR <1. This indicates that a large proportion of survival-related IRLs accounted for risk factors related to the prognosis of CRC.

### Establishment of IRLPI

**FIGURE 3:**
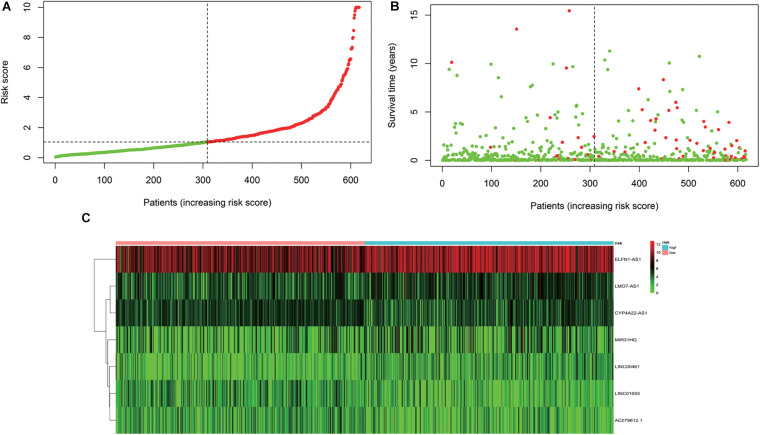
Development of the IRLPI. **(A)** The risk curve of each patient is reordered by the risk score. **(B)** Scatter plot of the patient survival curve. The red and green dots represent death and survival rates, respectively. **(C)** The heat maps show the signal expression profile of the high-risk group and the low-risk group. The pink bar is the low-risk group, and the blue bar is the high-risk group. The evolution from green to red represents an increase in gene expression.

According to the results of the multivariate Cox regression analysis, IRLPI was established. The risk score for each patient was calculated according to the following formula: risk score = [expression level of *LINC00461* × 0.2826] + [expression level of *LINC01055* × (−0.2275)] + [expression level of *ELFN1-AS1* × 0.3071] + [expression level of *LMO7-AS1* × 0.2742] + [expression level of *CYP4A22-AS1* × (−0.2466)] + [expression level of *AC079612.1* × (−0.1931)] + [expression level of *MIR31HG* × 0.1507]. Based on the risk scores, the patients were classified into high-risk groups and low-risk groups. Those with an IRLPI value greater than the median value were defined as high risk, whereas an IRLPI value lower than the median value defined as low risk. As displayed in Figure 3, the number of deaths in the high-risk group was significantly higher than that in the low-risk group.

### Assessment of IRLPI

**FIGURE 4:**
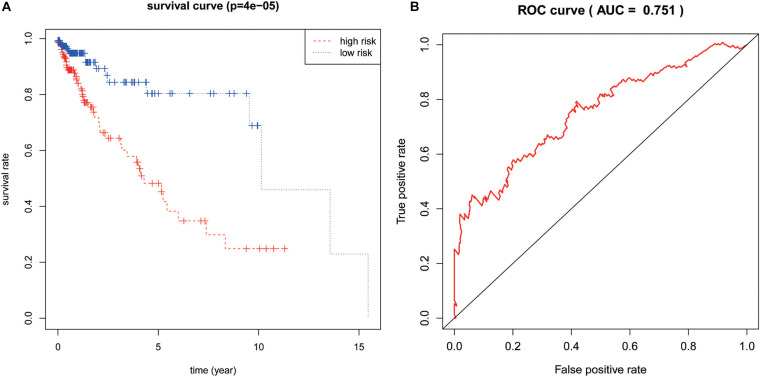
Assessment of IRLPI. **(A)** The K-M survival analysis is based on IRLPI. Patients with low-risk scores showed higher OS, compared to those with high-risk scores. **(B)** The survival-related ROC curve verifies the prognostic value of IRLPI.

The calculated IRLPI values were applied to predict the survival rate of CRC patients. As demonstrated in Figure 4A, the OS was higher in the low-risk group than in the high-risk group (*P* < 0.001), suggesting a better prognosis. The ROC curve was drawn and the AUC was calculated to evaluate the prediction accuracy of the established IRLPI (Figure 4B). The area value calculated is 0.855, indicating that IRLPI is of extraordinary value for the prediction of CRC prognosis. Besides, both results of univariate and multivariate analysis revealed a significant correlation between IRLPI and survival (Table 1). Therefore, IRLPI can be regarded as an independent predictor when multiple clinical parameters are considered.

**TABLE 1:** Univariate and multivariate analysis of CRC.

Variables	Univariate analysis		Multivariate analysis	
	HR (95% CI)	*P* value	HR (95% CI)	*P* value
Age	1.03(1.007–1.053)	0.0109	1.0358(1.001–1.072)	0.0439
Gender (male/female)	1.277(0.7719–2.112)	0.341	1.3798(0.6442–2.955)	0.4075
T stage	1.313(0.592–2.914)	0.503	1.6892(0.3569–7.995)	0.5087
N stage	1.821(1.09–3.044)	0.0222	0.3629(0.1091–1.207)	0.0982
M stage	4.251(2.406–7.51)	6.22E-07	0.4576(0.1443–1.451)	0.1842
Pathological stage	2.187(1.274–3.754)	0.00456	9.3175(1.5837–54.82)	0.0136
Tumor status (with tumor/tumor free)	2.964(6.842–54.86)	2.38E-08	28.2013(5.8972–134.863)	2.88E-05
IRLPI	1.201(1.141–1.264)	2.03E-12	1.10052(1.0144–1.194)	0.0212

*CRC, colorectal cancer; HR, hazard ratio; IRLPI, the prognostic index of immune-related lncRNAs.*

To further evaluate the clinical application value of the established risk scores, the correlation between the IRLs in IRLPI and clinicopathological factors were researched. As displayed in Table 2, tumor status and N stage were more related to IRLs in IRLPI. Furthermore, as an independent indicator, IRLPI had significant differences in tumor status and N stage, suggesting that IRLPI can accurately predict the N staging of CRC patients (*P* < 0.05). However, the IRLPI has no significant differences in age, gender, pathological stage, T stage, and M stage.

**TABLE 2:** The correlation between the expression of the IRLs and the clinical factors in CRC.

lncRNAs	Age (>68/ ≤ 68)	Gender (male/female)	Tumor status (with tumor/tumor free)	Pathological stage (III&IV/I&II)	T stage (T3–4/T1–2)	M stage (M1/M0)	N stage (N1–2/N0)
LINC00461	0.2743	0.3939	0.9923	0.8624	0.0722	0.7223	0.5493
LINC01055	0.7873	0.0431	0.0300	0.2680	0.1128	0.0286	0.6924
ELFN1-AS1	0.9712	0.5631	0.0039	0.7356	0.2659	0.1267	0.7576
LMO7-AS1	0.3229	0.7551	0.7640	0.0725	0.0258	0.0700	0.0202
CYP4A22-AS1	0.4912	0.6796	7.29E-08	0.4534	0.8477	0.4680	0.4739
AC079612.1	0.0582	0.5959	0.0146	0.2019	0.7068	0.4850	0.0793
MIR31HG	0.6382	0.3311	0.3108	0.4416	0.2903	0.8431	0.5400
IRLPI	0.1517	0.8538	0.0004	0.1434	0.0699	0.0619	0.0425

*IRLs, immune-related lncRNAs; CRC, colorectal cancer; IRLPI, the prognostic index of immune-related lncRNAs.*

### Mutation Landscape of IRGs in IRLPI

**FIGURE 5:**
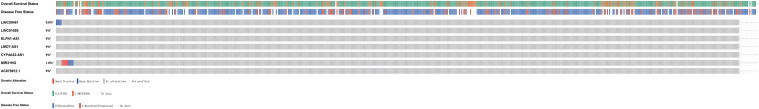
The landscape of genetic alterations of IRLs in IRLPI.

Based on the important clinical application value of IRLs, a comprehensive study of their molecular properties was conducted. In particular, the genetic variation of these lncRNAs was analyzed by the cBioPortal website. It was found that most IRLs showed little genetic variability, including *LINC00461* has 0.8% deep deletion, and *MIR31HG* has 1.8% amplification and deep deletion (Figure 5).

### Clinical Application of IRLPI

**FIGURE 6:**
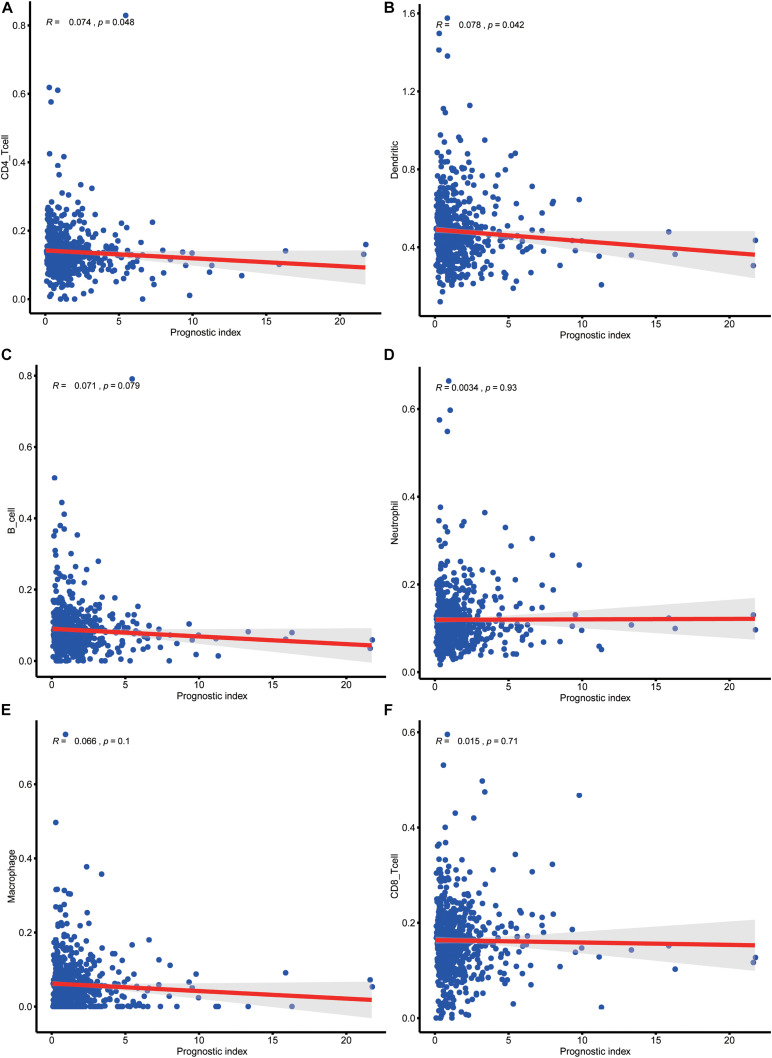
Analysis of the relationship between IRLPI and the abundance of immune cell infiltration. **(A)** CD4 T cells, **(B)** dendritic cells, **(C)** B cells, **(D)** neutrophils, **(E)** macrophages, **(F)** CD8T cells. *P* values are based on the Wilcoxon rank-sum test.

To evaluate whether IRLPI can exactly reflect the condition of the tumor immune microenvironment, the relationship between IRLPI and the abundance of immune cell infiltration was analyzed. The acquired results showed that IRLPI was significantly correlated with two types of immune cells, including CD4 T cells (Figure 6A) and dendritic cells (Figure 6B). However, there were no significant correlations between IRLPI and B cells (Figure 6C), neutrophils (Figure 6D), macrophages (Figure 6E), and CD8 T cells (Figure 6F).

### Verification of IRLs in IRLPI

**FIGURE 7:**
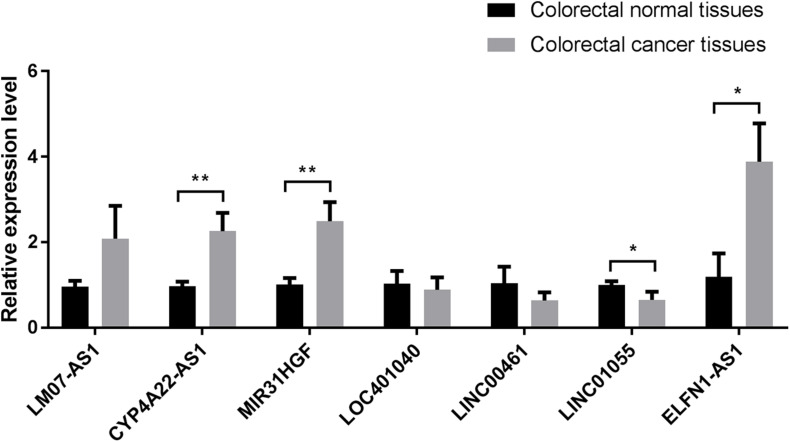
Comparison of relative expression of lncRNAs in IRLPI between colorectal cancer tissues and colorectal normal tissues. *P < 0.05, **P < 0.01.

To verify the reliability of IRLs in IRLPI, we collected ten clinical samples of colorectal cancer patients for qRT-PCR molecular verification. The results showed significant differences (*P* < 0.05) in the relative expression of four lncRNAs (*CYP4A22-AS1,MIR31HGF, LINC01055, and ELFN1-AS1*) between colorectal cancer tissues and colorectal normal tissues, as shown in Figure 7.

## Discussion

Colorectal cancer is a common malignant tumor of the digestive system, but the potential mechanism for CRC occurrence and development was not completely clear. At present, it is agreed that the abnormal immune response is closely related to the occurrence and development of cancer. On one hand, the immune system can recognize and destroy tumor cells, on the other hand, the tumor cells can evade the monitoring of the immune system through immune escape and immune-suppression (Leone and Powell, 2020). Studies have found that lncRNA is an important regulator of cancer immune response. Previous studies had shown that four immune-related lncRNAs including *BZRAP1-AS1*, *EMX2OS*, *ZNF667-AS1*, and *CTC-429P9.1*, significantly correlated with immune cell infiltration and had important prognostic value for cervical cancer patients (Zheng J. et al., 2020). Another study found that five immune-related lncRNAs containing *AP001007.1*, *LBX-AS1*, *MIR155HG*, *MAPT-AS1*, and *LINC00515*, regulate the tumor immune microenvironment through the interaction of cytokines and cytokine receptors, complement and coagulation cascades to affect the prognosis of glioma (Wang et al., 2020). Emerging evidence had indicated that immune-related lncRNAs (IRLs) can be used to characterize the infiltration of immune cells in tumors, are potential targets for cancer treatment, and have predictive value for survival and prognosis (Denaro et al., 2019). Therefore, it is important to identify effective biomarkers of CRC.

In recent years, gene signatures developed by the combination of high-throughput sequencing technology and bioinformatics have been widely used in individualized therapy and prognosis evaluation, and the prediction accuracy is better than a single biomarker (Yu et al., 2018). Therefore, it is necessary to establish immune-related lncRNA signatures for the treatment and prognosis of patients with colorectal cancer.

In this study, 240 differentially expressed IRLs were screened from CRC tissues and normal tissues by used the bioinformatics method. Eight IRLs including *LINC00461, LINC01055, ELFN1-AS1, LMO7-AS1, CYP4A22-AS1, AC079612.1, LINC01351*, and *MIR31HG* were identified by univariate COX regression analysis, and most of them were positively correlated with poor prognosis of CRC. It is reported that *LINC00461* played vital roles in a variety of biological processes, including cell migration, cell invasion, and cancer progression (Yu et al., 2019). Studies reported that *LINC00461* played an oncogenic role in CRC cells through the NFIB signaling pathway, and mediated cisplatin resistance of rectal cancer by targeting the *miR-593-5p*/*CCND1* axis, showing that *LINC00461* may be a prognostic biomarker for CRC (Qu et al., 2020). High expression of *LINC01055* was associated with a better prognosis of CRC patients, whereas high expression of *ELFN1-AS1*was associated with a poorer prognosis (Sun et al., 2020). Based on survival analysis, it was found that high expression of *DUXAP8* and *ELFN1-AS1* predicts poor prognosis in colon cancer (Chen et al., 2020). Zheng et al. found that high expression of *AC135178.1, LINC00535*, and *LMO7-AS1* correlated with poor survival in patients with childhood kidney cancer (Zheng H. et al., 2020). Cheng et al. identified that the expression profiles of 3 lncRNAs by using a LASSO regression model, including *CYP4A22-AS1, AP000695.6*, and *RP11-108M12.3*, were significantly related to the prognosis of gastric carcinoma (Cheng, 2018). Wang et al. (2018) found that 15 lncRNAs, including *AC079612.1*, may act as ceRNAs to play a crucial role in the modulation of cancer-related pathways. Tu et al. (2020) demonstrated that *MIR31HG* overexpression predicted unfavorable OS in lung cancer, while predicted better OS and disease-free survival for gastrointestinal cancer patients.

The incidence and clinical characteristics of colorectal cancer, such as pathological classification, tissue type, and location, are significantly different in patients, and the prognosis of patients with the same stage is also different. Therefore, it is necessary to study more about the prognostic factors of colorectal cancer. We developed IRLPI using multivariate COX regression analysis and divided patients into low-risk groups and high-risk groups based on risk scores. The survival time of patients in the low-risk group was significantly longer than in the high-risk group. The reliability and validity of the index in predicting the prognosis of CRC were confirmed by ROC curve analysis. To evaluate the feasibility of IRLPI, the correlation analysis between clinicopathological factors and risk scores showed that IRLPI is correlated with tumor status and N staging, and can be used as a useful supplement to TNM staging.

The tumor microenvironment is composed of tumor cells, interstitial cells, capillaries, microlymphatic vessels, tissue fluid, numerous cytokines, and a small amount of infiltrating cells. It plays an important role in tumor occurrence, development, and prognosis. By changing or reversing the interaction between tumor cells and the immune system in the tumor microenvironment, and then destroying the tumor cells in the primary tumor and metastasis, we may achieve successful immunotherapy. The evaluation of the correlation between IRLPI and immune cell infiltration can provide evidence for changing the state of the tumor microenvironment. Our results show that IRLPI is significantly associated with CD4 T cells and dendritic cells in CRC patients. These immune cells play important roles in the prognosis of CRC. Sasidharan et al. identified a “poor prognosis CD4 gene signature” (ppCD4sig), found that patients with high ppCD4sig score showed shorter disease specific survival and progression-free interval, which provided novel insights and a unique prognostic gene signature of CD4 + tumor-infiltrating lymphocytes in the CRC microenvironment (Sasidharan Nair et al., 2020). Lavoie et al. reported that loss of free fatty acid receptor two promoted colon tumorigenesis in mice by reducing gut barrier integrity, increasing tumor bacterial load, promoting exhaustion of CD8 + T cells, and over activating DCs, leading to their death (Lavoie et al., 2020). Liu et al. researched the association of dendritic cell-specific intercellular adhesion molecule 3-grabbing non-integrin (DC-SIGN) macrophages with clinicopathological parameters, found that DC-SIGN + macrophages were associated with immune invasive tumor-associated macrophages and indicated poor prognosis and inferior therapeutic responsiveness to fluorouracil-based adjuvant chemotherapy (Liu et al., 2020). In summary, dendritic cells are closely related to the prognosis of gastrointestinal tumors.

Finally, we used qRT-PCR to perform molecular verification of the seven IRLs in IRLPI. The expression levels of four IRLs in IRLPI (*CYP4A22-AS1,MIR31HGF, LINC01055, and ELFN1-AS1*) were significantly different between colorectal cancer tissues and colorectal normal tissues. The other three IRLs did not have significant differential expression. The reason for this problem may be caused by the very small clinical sample size.

Compared with previous studies, we were the first to use immune-related lncRNA to construct a risk assessment model for colorectal cancer, and we also collected clinical samples to verify the lncRNA in the risk assessment model. In this study, we only collected ten clinical samples, which limited the verification of lncRNA. In future research, we plan to collect more clinical samples to study the pathogenesis of colorectal cancer.

In conclusion, based on immune-related lncRNA and the survival status of patients, we successfully constructed a prognostic model with a powerful predictive function for colorectal cancer. There are significant differences in the immune status of patients in the high-risk group and the low-risk group, which may become an indicator for evaluating whether they are suitable for immunotherapy. The establishment of IRLPI provides convenience for the choice of clinical CRC treatment. Although we have verified clinical specimens, as an exploratory study, its application value still needs to be further verified by multi-center large sample clinical research.

## Data Availability Statement

Publicly available datasets were analyzed in this study. This data can be found here: https://portal.gdc.cancer.gov/ and http://bio-bigdata.hrbmu.edu.cn/ImmLnc/.

## Ethics Statement

The studies involving human participants were reviewed and approved by the Ethics Committee of the Jiangsu Provincial Hospital of Integrated Traditional Chinese and Western Medicine. The patients/participants provided their written informed consent to participate in this study.

## Author Contributions

FQ, HX, GW, YJ, JY, CH, and CY: conception. HX, YM, JQ, and LL: data collection and analyses. FQ, HX, and JH: writing, review, and revision of the manuscript. HX, LL, and JH: study supervision. All authors contributed to the article and approved the submitted version.

## Conflict of Interest

The authors declare that the research was conducted in the absence of any commercial or financial relationships that could be construed as a potential conflict of interest.

## Supplementary Material

Supplementary Table 1 Detailed information regarding qRT-PCR primer of IRLs in IRLPI.
